# Social, environmental, and COVID-19 pandemic-related effects on women's food security and health in Honduras

**DOI:** 10.1007/s43545-022-00448-y

**Published:** 2022-09-09

**Authors:** Ingrid Fromm, Andrea Reiche, Dilcia Sauceda, Elena Rivera

**Affiliations:** 1 School of Agricultural, Forest and Food Sciences, Bern University of Applied Sciences, Zollikofen, Switzerland; 2 Programa Posgrado en Agricultura Tropical Sostenible, Universidad Zamorano, Zamorano, Honduras; 3 Departamento de Salud Pública, Facultad de Ciencias Médicas, Universidad Nacional Autónoma de Honduras, Tegucigalpa, Honduras; 4 Departamento de Nutrición, Facultad de Ciencias Médicas, Universidad Nacional Autónoma de Honduras, Tegucigalpa, Honduras

**Keywords:** Gender, Food systems, Nutrition, COVID-19, Honduras

## Abstract

The COVID-19 pandemic has disproportionally affected women in Honduras in terms of loss of employment and income opportunities, access to healthcare services, and increased poverty and food insecurity. The pre-pandemic gender inequalities in Honduras have resulted in harsher conditions for women since the onset of the pandemic. Early reports indicate that women have lost employment and incomes and have been burdened by other effects of the pandemic, such as more household work, childcare activities, and home schooling. Marginal groups such as indigenous women face greater challenges because of the structural and systemic inequalities which have existed for a long time. The impact of the COVID-19 pandemic has also differed across geographic areas and between rural and urban settings. In addition to the pandemic, the economic outlook for women in Honduras has worsened since the impact of Hurricanes Eta and Iota in November 2020, which displaced over a million people. The agricultural sector was devastated, and infrastructure was severely damaged. The recovery efforts have been slow because of the COVID-19 pandemic. This paper explores the root causes of gender inequalities and how it affects women’s food security and health.

## Introduction

The COVID-19 pandemic has disproportionally affected women in Honduras from a public health, food security, and socio-economic perspective. Although the pandemic has been primarily a health crisis, there are detrimental economic, social, and political effects. As nationwide lockdown measures were implemented in March 2020, the existing inequalities afflicting women became more evident. Estimates published by UN Women (2020) reveal that the pandemic will leave 118 million women and girls in poverty in Latin America and the Caribbean. This is due to the reduction in economic activity which primarily affects women, particularly women employment in the informal sector who cannot claim social benefits. As women have lost their livelihoods when containment measures were implemented to slow the spread of the novel coronavirus in March 2020, they faced the direct impact of these measures. No social services, employment benefits or other programs could cushion the impact of a loss of wage or replace a daily income, especially for women employed informally. In Latin America, more than half of women work in sectors affected by the COVID-19 pandemic, such as domestic work, service, commerce, manufacturing, tourism, and primary care activities. These are the economic sectors where women are over-represented in the first line of response, but with minority participation in decision-making and with no safety net in the case of a loss of employment (UN Women 2020). According to a report published by CARE International (2020), the COVID-19 pandemic will precipitate the most severe declines in human development since the Human Development Index was introduced in 1990. In Honduras, vulnerable populations, including women, indigenous people, and others in a situation of poverty, are hardest hit. A recent report published by CEPAL (2021) states that the pre-existing inequities for indigenous women in Latin America, means that they are disproportionately affected by the pandemic, as access to health is limited and up to 80% of indigenous women in the region are employed in the informal sector.

One natural consequence of economic downturn and loss of employment opportunities is increased food insecurity. As Swinnen and McDermott (2020) point out, the impact of Covid-19 pandemic on global food security is also heterogenous. Poor and disadvantaged groups, including women, are more likely to suffer the consequences of food and nutrition insecurity than people with more economic resources. Because of increasing food prices and loss of incomes, disadvantaged groups are less able to adjust to the effects of the pandemic, as they are less likely to have any additional assets or savings and rely on daily work and wages to pay for food. Swinnen and McDermott (2020) also point out that developing countries have fewer fiscal options to respond to the economic fallout from Covid-19 pandemic and to finance public spending on programs like cash transfers and safety nets for disadvantaged groups. The access and provision of healthcare is often limited. Basic infrastructure such as proper water and sanitation facilities are basic and important in protecting the population against COVID-19. Low-income communities in urban areas with limited access to water, as is the case of Tegucigalpa, have reported a rapid spread of COVID-19, thus placing low-income people, particularly women, in a more precarious situation.

Over the past two decades, the proportion of undernourished population in Honduras dropped from 22% in 2000 to approximately 14% in 2020 (GHI 2021). However, Honduras has faced not only the effects of the pandemic which has trigged an economic downturn, but also the impact of two severe weather events in November 2020—the impact of Hurricanes Eta and Iota. The 2020 Atlantic hurricane season was extremely active and devastated Central America. Hurricanes Eta and Iota, which brought severe flooding, landslides and destroyed homes and infrastructure, displaced an estimated 7 million people in the region, about 1.5 million people in Honduras have lost homes and livelihoods (ReliefWeb 2020). People affected by floods, who lost their homes, and all their belongings were automatically placed in a food insecure situation, relying mainly on donations and humanitarian relief to be able to feed themselves and their families. The dire economic outlook prompted by the COVID-19 pandemic has further deteriorated with the passing of these two devastating hurricanes. As people urgently sought shelter and tried to evacuate the Valle de Sula and other regions in northern Honduras, the physical distancing, and other recommended precautions to minimize the spread of COVID-19 became a second priority. An accelerated infection rate among people who were crammed into temporary shelters with little or no sanitary facilities, or the possibility of physical distancing was a consequence (ReliefWeb 2021).

As Heady and Ruel (2020) poignantly state, the COVID-19 pandemic has all the makings of a perfect storm for global food security and malnutrition. The COVID-19 pandemic has exposed the imperfections and inequalities in the global food systems and amplified the existing weaknesses. It has affected how people grow, buy, transform, prepare, and sell agricultural products and food (CARE 2020; Reardon et al. 2020). The nutritional status of vulnerable groups, such as women in disadvantages settings, can lower through multiple mechanisms. One mechanism stems from an income loss and loss of ability to purchase food, especially nutritious food. When lockdown measures were set in place in Honduras, many women in middle to lower-income settings increasingly lost the ability to process and sell food. Malnutrition can also be exacerbated through the inability of governments to provide viable alternatives to mitigate the effect of the pandemic measures, such as food packages or supplementation of food to those facing food shortages. Although the Honduran government has social safety nets and provided cash transfers to the most vulnerable groups in urban areas, the COVID-19 pandemic required additional resources to mitigate the economic effects of the lockdown measures which were in place from March until June 2020. Through the Ministry of Development and Social Inclusion, vulnerable groups in 90 municipalities received food assistance for four months and approximately 250,000 independent workers received a single cash transfer to mitigate some of the effects of the pandemic lockdown measures (SEDIS 2020). The World Bank (2020) estimates that the GDP in Honduras contracted by 7.1% in 2020, thus, the government’s response falls short of having the expected effect, as lower- and middle-income families struggle to cope with the effects of the pandemic and natural disasters. Another socio-economic effect of the pandemic was the lower inflow of remittances to Honduras, due to GDP contraction in the United States of America, from where many migrant workers send money to their families in Honduras. According to the Interamerican Development Bank (2016), 69% of the remittances recipients in Honduras are women, so as the cash inflow decreased, women were placed in a more vulnerable situation. This same report states that 58% of these women are also the heads of households, so the burden of coping with a loss of cash flow, household and primary care responsibilities and limited employment possibilities has been harsh.

The objective of this paper is to analyze how the pandemic has affected women in terms of food security and access to food. In particular, the case of more vulnerable populations, in this case the Lenca indigenous group of Western Honduras was examined to explain which factors place women in a marginalized population at a higher risk of not being able to cope with the impact of lockdown measures or a sudden loss of employment. This analysis sheds light on structural issues which limit the access of women to key services and benefits which could help them become more resilient. The findings contribute to a growing body of empirical evidence about gender and inequality, particularly in rural areas and could support policymakers to draft better policies and design programs with the goal of reducing the inequalities among women and vulnerable groups.

## Methods

To assess how women have been impacted in the first months of the COVID-19 pandemic, primary data on health, household income, and employment was collected. An online household survey was conducted to assess the economic impact of the lockdown measures. Data were collected from 28,000 households in three different phases: at the beginning of the lockdown (April 2020), a month later (May 2020), and a third follow-up survey was conducted in December 2020, after the lockdown measures had been lifted. A descriptive analysis of the household situation in these three phases was conducted. Socio-economic factors were reviewed to understand how households and in particular women, were affected in terms of employment and change in income.

Additional data from secondary, official sources presented by the Ministry of Health and the Communications Office of the government of Honduras was analyzed to understand the scale of the pandemic and the effect on women in Honduras. These statistical figures on infection rates and household affected highlighted how the pandemic was affecting households. Finally, results of a case study on indigenous rural women in western Honduras is analyzed in this paper to understand some of the gender disparities and struggles of women before and during the pandemic. To finalize the discussion on gender disparities in the face of the COVID-19 pandemic and the effect on food security, nutrition and health, policy options based on the evidence collected are presented.

## COVID-19 epidemiological impact on women

The first and second waves of the COVID-19 pandemic were challenging for Honduras, By the end of 2020, the rate of positive cases per 100,000 inhabitants in Honduras was 32. The age range with the highest number of infections was the 30–39-year group, however the fatality was higher in the range of 70–79-year-old people, men being most affected both in morbidity (52%) and lethality (64%). Health care workers constitute one of the most affected sectors, representing 8.6% of all cases, with 73% of the reported cases being women in the 30–39 age range. The professional profile most affected is the nursing staff (Despacho de Comunicaciones 2020).

Access to the health care system in rural areas in Honduras was challenging. Lower resources and the centralization in the provision of healthcare services, usually located in larger cities (Melvin et al. 2020) limited the access to health care services in rural areas. The indigenous population in Honduras is particularly underserved. The pandemic has caused not only a health crisis, but also an economic one, causing a decline in the country's economy. Thus, access to healthcare, which to a large extent is paid out-of-pocket, has become more restricted for people, especially after lockdown measures were adopted, forcing a large portion of the population to lose their incomes. Most workers in Honduras are employed informally, according to Hernandez Ore et al. (2016) only a fifth of the economically active population is employed formally. While Honduras has a comparatively higher minimum wage and benefits to other countries in the region, most of the people do not have access to these benefits because of informal employment. As lockdown measures were instituted, people employed informally could not make an income and had no safety net in the form of unemployment benefits to compensate for a loss of income. While the Honduran government’s intervention to minimize the economic impact of COVID-19 did focus on maintaining the livelihood of households during the crisis, and fiscal measures to reduce pressure on firms, the policies benefited registered, formal sector small and medium enterprises (UNDP 2020).

According to the World Bank (WB 2020), 48% of the population of Honduras lives in poverty, with one of the highest inequality rates in the world. In November, the country was hit by hurricanes ETA and IOTA, leaving damage to the agricultural, livestock, and industrial sectors, in addition to damage to housing and road infrastructure. Employment rates fell, estimating a decrease of − 9.4%, which represents losses of around 400,000 jobs (UNAH 2020a, b).

## Disruptions in the food system, food and nutrition insecurity and gender

In February 2019, a set of government strategies to strengthen food security and nutrition was approved. The SAN National Policy and Strategy with a horizon of 2030 (PyENSAN 2030) laid out the strategic framework to improve and strengthen this area of national development. In this document, nutritional food is defined as the provision of timely and permanent access to the food people need, in quantity, quality and acceptable form for its proper consumption and use, guaranteeing people its full human development. Given that Honduras is a developing country, with 36% of the population living in extreme poverty, guaranteeing sufficient and sustainable food is key to improving poverty and food insecurity rates.

According to food security reports, published in December 2019, 18% of the Honduran population was in phase 3 (people in crisis), which required urgent action because of food insecure. In this same document, a projection was made in which estimated that from March to June 2020, this percentage would increase to 24% of the population. Unfortunately, the effects of COVID-19, pushed the percentage of food insecure people to 32% of the population. In addition to this precarious condition, the situation continues to be exacerbated by the catastrophic effects from the pandemic and the recent impact of two severe hurricanes which hit Honduras in November 2020. According to the report carried out in 13 of the 18 departments^1^ of Honduras, the CIF concluded that in all the departments, an increase in the population in Crisis condition (Phase 3) was observed, increasing from 19 to 32% of the population in a food insecure condition. Therefore, the 13 departments of Honduras were classified in a Crisis Condition, which implies that households are not able to meet their food needs without depleting essential assets of their livelihoods.

The main causes of this increase in food insecurity were the lockdown measures implemented by the Government of Honduras, such as the restriction in the movement of people, products, and transportation, directly affecting the agri-food chain. In addition, this situation was worsened by the rise in food prices due to low food production and loss of income, either due to job loss or decreased receipt of remittances. Women were disproportionally affected by job losses and a lower inflow of remittances (UN Women 2020).

To analyze the impact of the crisis on Honduran households, the Institute for Economic and Social Research (IIES) of the National Autonomous University of Honduras carried out three rapid assessments, with the purpose of obtaining data on the impact of COVID-19 on the family economy. The first phase of the study included 28,000 households in all 18 departments. Respondents were contacted by UNAH students and were sent a link to answer the online survey by mobile phone or e-mail. The results indicated that in 37% of the homes, no one worked. Furthermore, a decrease in income was reported in 54% of the surveyed households. In these households, the income had decreased by up to 60%, thus increasing the food insecurity situation of the families.

The second phase of the study was carried out in May 2020. In this study, 10,023 people in all 18 departments of the country were sampled. A triangulation of the data was done with the previous study. Results indicated that 56% of the respondents reported a decrease in income by April 2020 and 68% reported a decrease in income a month later, in May 2020. In the first study, 89.2% of households had one or more working members, while, after the second study, only 74.46% of households had one or more working members. The reduction in the employment of these households was approximately 32%. In addition, there was an increase in the percentage of families purchasing their food on a weekly basis to doing so biweekly, which could be due to the decrease in family income.

In the third study follow-up study, conducted in December 2020, 12,684 households were sampled. The results showed that the percentage of job recovery during the COVID-19 pandemic was close to 20% for the people who were laid off from March to December. The tight lockdown restrictions were lifted in the second half of 2020, thus allowing people to return to work, open business, and allowing the free circulation of people, which is of essence in economies where a large proportion of the population is employed in the informal sector. Similarly, 56% of the suspended employees managed to rejoin the activities they engaged in prior to the pandemic. Nevertheless, 51.6% of households indicated that the income had decreased since the start of the pandemic. Only four out of every ten households maintained their income level and only 4.4% reported an increase in their income. Even though there was an improvement in income in the last quarter of the year in several of the surveyed households, at least half of the surveyed households reported that they had to withstand the negative economic effects of the pandemic and the natural disasters which displaced over 1 million people in northern Honduras.

The World Food Programme (2020) monitored the situation of SAN Food and Nutritional Security in Honduras using its Vulnerability Analysis and Mapping (VAM) tool. This assessment was carried out in coordination with government and non-governmental organizations and associations. The evaluation sample consisted of 6,183 households in the 18 departments of the country, with 81% of the respondents in rural areas and 19% in urban areas. From those surveyed, 49% were female and 51% male. The average household size was 4.6 members. In rural areas, the number was higher. The results indicated that prior to the pandemic, the average number of employed family members in the household was 1.2, but this number dropped to only 0.59 who had a job after the onset of the COVID-19 pandemic, per household. This situation led to a worsening of food insecurity with 17% of those surveyed selling their productive belongings to be able to buy food and pay for other basic necessities. Only 11% of the respondents reported having enough basic grains like beans and corn available for the next harvest and 89% of those surveyed had food reserves for the next month.

One critical finding is the relation of low income and access to food. The respondents have reported that 75% of their income or more is used to purchase food. In 79% of the rural households and 83% of urban households, people had less income to buy food. In addition, 61% of those surveyed reported purchasing food using their savings or credit. The lack of work caused the population to become indebted to satisfy their basic needs. Regarding food consumption, 51% of those surveyed said they were reducing the amount of food and the number of meals to face the crisis and 9% indicated that they were consuming foods that are not of their preference as a coping measure.

## COVID-19, food and nutrition insecurity and gender: the case of indigenous women in Intibucá, Honduras

The results of the household survey revealed that a large proportion of the households in Honduras faced adverse effects from the pandemic. One guiding question of this study was if women in marginalized communities face more adverse effects. To understand structural and systematic issues, we examined the Lenca indigenous community, the largest ethnic minority in Honduras. According to the World Directory of Minorities and Indigenous Peoples (2020), there are approximately half a million Lencas, living in 612 different villages and towns, mostly located in the mountainous western part of Honduras. Their social structure is strong and defined by men participating mostly in agriculture and women engaged in traditional crafts such as weaving and pottery, which generates an additional household income. Nevertheless, there are gender disparities emanating from the defined roles, where the man works outside the home and women take over household tasks and child-rearing.

Different NGOs, among them the Asociación de Mujeres Intibucanas Renovadas (AMIR), work together with women to improve their living condition. AMIR is a grassroots organization that works exclusively with local women. AMIR currently has 650 members in different communities in the department of Intibucá, which is in the western area of Honduras. Their interventions have positively impacted the lives of many households, by providing a space where women are heard, but also helping women develop strategies to face the ideological and structural barriers to which they have been subjected. This includes better access to knowledge, economic empowerment, and participation in decision-making. Likewise, through the involvement of other members of the household and the recognition of women’s rights, important advances have been made in the transformation of gender roles that place women at a disadvantage.

In collaboration with the Asociación de Mujeres Intibucanas Renovadas (AMIR) in the department of Intibucá, a qualitative study was carried out using the participatory photovoice research-action methodology (Wang 1999). The methodology facilitated the understanding of the main problems related to rural agriculture and the contribution of women in sustainable agriculture (Reiche 2020). The study was conducted in 2019, before the onset of the pandemic, but the effects of the pandemic on the community has since been documented.

In Intibucá, 60% of the population is dedicated to agriculture (INE 2013). According to the latest Report on Food and Nutrition Security Indicators (UTSAN 2019) the average number of members per family is five people, the highest in Honduras. In addition, 100% of families plant maize and beans and about 52% of them have about 3 ha of land, so more than 30% do not have basic grain reserves for more than three months (UTSAN 2019). The research findings showed that family agriculture and the production of horticultural crops of high commercial value represent the main source of food and income in rural households in Intibucá. However, by depending on it to guarantee their livelihoods, the problems associated with the increase in pests, extreme climatic events (droughts, floods, strong winds, and frosts), the lack of road and transport infrastructure, the dependence on intermediaries and market price variability, among others, have placed farmers in a highly vulnerable context.

Maize is the most important basic grain in the daily diet of Intibucá households. It is used in various dishes as a staple, and it is part of the Lenca cultural identity. For this reason, all the households in the rural area of Intibucá grow maize. When the demand for self-consumption is satisfied, the income generated when grains are sold is used for other basic needs that improve their quality of life. Among them, access to services such as health, education and transportation, the acquisition of material goods, the purchase of seeds or the raising of domestic animals such as chickens, swine, and cows. Owning animals promotes the diversification of the diet and the generation of income from the sale of by-products, which, in turn, allows the purchase of other foods and inputs. This small-scale farming system where maize and bean reserves are kept in storage (i.e., silos) is a monetary reserve mechanism, since the grains are sold in times of crisis, food shortages or unforeseen events, such as the COVID-19 pandemic.

On the contrary, when maize yields are low, the households face difficulties. This scenario is the most frequent and impacts men and women in a different way. For women, not having enough maize for daily consumption is one of their biggest concerns. Maize availability is intrinsically linked to their daily life as they are responsible for preparing food for the family as part of traditional gender roles.

Consequently, households rely on different strategies to cope with changes in maize yields and availability throughout the year. They generally choose to reduce the daily consumption, sell domestic animals and material goods, exchange food or labor for maize, and ultimately, migrate to other areas or even abroad in search of sources of employment. In the case of farmers who are engaged in cash crops, the loss due to low productivity, low prices, and the lack of access to stable markets, caused great economic losses. The resources to establish subsequent planting cycles, access to services and the purchase of food for consumption, including maize, have also decreased. In small-scale agriculture in this region, there are family gardens, which are located near the home. Generally, gardens are small areas in which some farm animals, fruit trees, edible, ornamental and medicinal plants, and herbs coexist. Although no extensive care or financial resources are needed, the time available for women to work in it is usually limited. This is one of the coping mechanisms which have helped the community remain resilient during the COVID-19 pandemic and the lockdown measures.

Despite the limitations, AMIR has managed to integrate the agroecological approach for the sustainable production of the home gardens, seeking to reduce the use of agrochemicals and the use of local resources. Through training and skills development processes, several women have transformed the gardens into a place of empowerment and income generation. AMIR's intention has been to promote vegetable garden production to diversify the diet, cushion periods of scarcity, and reduce the risk of food insecurity.

With mobility restrictions in place due to the Covid-19 health crisis and social distancing, it is possible that the outreach of AMIR has been weakened. When markets were closed, the possibility of transporting goods and accessing markets for the commercialization of agricultural products was minimized, reducing a secure source of income for women. The long-term effects of the loss of income among Lenca women is yet to be studied, but it is likely that whatever advancement the women in this study had achieved, will be reversed.

## Gender relations in agriculture and the COVID-19 pandemic

In Intibucá, most of the women in rural areas are engaged in agricultural work from a very young age. However, very few women ever achieve decision-making power in the home and manage resources. Their labor in the field is considered an extension of domestic tasks and, thus, it is not paid. Furthermore, the persistence of the inheritance system in rural areas gives preference to male descendants. Women who actively participate in agricultural work generally do not have the right to land or receive direct benefits.

Because agriculture is generally led by men, women do not participate in training or occasionally exclude themselves, expressing that men can perform agricultural tasks better than they can. If for any reason men are present in groups or decision-making circles, women do not express opinions, either because they are not considered or for fear of rejection or ridicule. This denotes the devaluation of the participation of female labor force and the exclusion that women experience. In addition, the marketing channels for crops of high commercial value are managed by men, who know the market better and decide which crop to plant. Whilst women are only dedicated to production work and informal marketing, their productive role is practically invisible, even when it is as important as a man’s. This represents a disadvantage for women who wish to enter these types of markets.

Traditional division of labor structures assigns multiple roles to women. The women work in the fields and in the garden, carry out most household chores, participate in community and religious activities. For this reason, the daily work of women begins very early and extends until the end of the day. On the contrary, men dedicate themselves exclusively to field work and rarely engage in household activities. These strenuous hours restrict women's time availability and even prevent them from pursuing other personal interests. The COVID-19 pandemic and the restrictions limiting the movement of people placed an additional pressure on women in the household. According to a report from UN Women (2020), indigenous women in Latin America face some of the most adverse consequences of the COVID-19 pandemic, as they lose jobs in the informal sector, the service sector (such as domestic work) and entrepreneurship opportunities. At the same time, the overload of unpaid work is and will likely remain, a major obstacle to future empowerment and autonomy.

## Policy options to minimize the impact of COVID-19 on women

Minimizing the negative impact of the COVID-19 pandemic requires change which addresses the inequalities which already place women in Honduras in a vulnerable situation. The High-Level Panel of Experts on Food Security and nutrition (HLPE), governments need to consider the broader interactions with food security and nutrition in their action plans, to minimize the impact of the pandemic (CFS 2020). Although the right number of resources should be allocated to public health measures, ensuring food security and nutrition of the general population requires a substantial amount of resources as well. Plans also must respond to the needs of different communities and groups, including women and vulnerable groups, such as indigenous communities with more limited access to resources.

According to a report by UN Women (2020), the Latin American region needs to address gender equality for a sustainable recovery from the pandemic. The pre-pandemic gender inequalities such as the low female labor force participation is likely to result in a more negative impact for women. The female labor force participation rate (46%) is lower than both the male rate, which is estimated to be 85%. By comparison, the female rate labor force participation for the Latin American and Caribbean region is 53% (Hernandez Ore et al. 2016). Limited employment opportunities available to women, who face higher unemployment rates and lower average wages has worsened since the beginning of the pandemic. Not only is female participation in the labor force a limiting factor, but their higher participation in the informal sector also means that one of the most important social benefits—access to healthcare is therefore limited. In Honduras, access to the National Institute of Social Security (Instituto Hondureño de Seguridad Social), which provides access to health care and is the public provider of pension funds, is only accessible to those employed in the formal sector. The needs of women should be considered in the plans devised to mitigate the impact of the pandemic and promote a sustainable and fair recovery.

One proposition is to strengthen the financing of policies focused on women during and after the pandemic. These include the provision of comprehensive services in gender violence, sexual and reproductive health and care, and a basic emergency income for women in poverty. In Honduras, the National Institute for Women reported a 14% increase in the domestic violence reports from February to May 2020 (INAM 2020). The recommendations also address socio-economic protection programs such as the formalization, remuneration and social security of workers, as well as care workers, which in the case of Honduras, are mainly women. UN Women (2020) estimates that domestic work accounts for between 14.3% and 10.5% of women's employment in the Latin American region and more than 77.5% operate in the informal sector. In Honduras, as lockdown measures were implemented, a significant number of women working informally as domestic employees or in the service sector, without contracts and without access to social protection, were sent home and thus lost wages for several months. By implementing social protection measures, women employed in the domestic and service sectors could mitigate the negative impact of a loss of wage. In addition to strategies to help women employed informally in the domestic and service sectors, initiates to promote women entrepreneurship are equally as important. Women entrepreneurs, from micro-enterprises to larger businesses generating employment, are more likely to have autonomy over the money they generate and are thus less likely to suffer from a complete loss of income.

In terms of food security, more support to food systems and supply chains will be needed to increase resilience. Not only should issues such as the safety of workers in the front line of food production be tackled, but other structural issues related to agriculture in Honduras should be addressed. The agricultural sector in Honduras is already in a vulnerable position because of the country’s geographic location, prone to natural disasters such as tropical storms and hurricanes such as Iota and Eta which completely devastated the agricultural sector in November 2020. Honduras is also part of the Central American dry corridor, which makes agricultural production, increased productivity, and competitiveness more challenging (Fromm 2020). The common government strategies to support the agricultural sector such as subsidies for inputs to maintain and increase agricultural production are important, but not enough in the face of the current challenges.

## Conclusion

Although the long-term effect of the COVID-19 pandemic remains to be seen, the information gathered thus far indicates that women in Honduras have been disproportionally affected in terms of loss of employment and income opportunities, access to healthcare services, and increased poverty and food insecurity. The pre-pandemic gender inequalities in Honduras have resulted in harsher conditions for women since the onset of the pandemic. Not only have more women lost employment and incomes, but additional burdens have been placed on them, such as more household work, childcare activities and even home schooling. Women in the informal sector have been greatly affected as they were the first to lose employment. People affected by the devastation of Hurricanes Eta and Iota faced food insecurity, relying mainly on donations and humanitarian relief to be able to feed themselves and their families for months. Marginal groups, such as indigenous women face greater challenges coping with unforeseen events such as the pandemic and the climate events of 2020 because of the systemic inequalities. Policies addressing these inequalities must be pursued so women are no longer placed in a more vulnerable situation. However, cultural and social structure issues also need to be addressed at the grassroots level, as the case of the Lenca women clearly demonstrates.

The impact of the COVID-19 pandemic has also differed across geographic areas and between rural and urban settings and these differences can give an indication as to what strategy can be put in place to make sure the correct measures are taken. Not only has the pandemic worsen the economic outlook, but the impact of Hurricanes Eta and Iota at the end of 2020 will have a long-lasting effect. The agricultural sector was devastated, and infrastructure was severely damaged. The recovery efforts have been slow because of the COVID-19 pandemic. Food security, nutrition and health are intertwined issues that affect women. The best alternative in the face of the COVID-19 pandemic is to address them as a whole and not as individual problems.

## Data Availability

The datasets generated and/or analyzed during the current study are not publicly available but may be made available from the corresponding author upon request.

## Footnotes

1 A “department” is the administrative division in Honduras, equivalent to a province or state.

## References

[CR102] CARE (2020) Preparing for the food and nutrition security impacts of COVID-19 chairs summary, preparing for the food and nutrition security impacts of COVID-19 webinar. May 26, 2020

[CR1] CFS (2020) Interim issues paper on the impact of COVID-19 on food security and nutrition (FSN) by the High-Level Panel of Experts on Food Security and nutrition (HLPE). Rome, High Level Panel of Experts of the Committee on World Food Security. http://www.fao.org/fileadmin/templates/cfs/Docs1920/Chair/HLPE_English.pdf

[CR2] CEPAL (2021) The impact of COVID-19 on indigenous peoples in Latin America (Abya Yala): between invisibility and collective resistance”, Project Documents (LC/TS.2020/171), Economic Commission for Latin America and the Caribbean (ECLAC), Santiago, Chile.

[CR3] Cruz MP, Santos E, Cervantes MV, Juárez ML (2020) COVID-19, una emergencia de salud pública mundial. Revista Clínica Española. https://www.ncbi.nlm.nih.gov/pmc/articles/PMC7102523/10.1016/j.rce.2020.03.001 PMC7102523 32204922

[CR4] Despacho de Comunicaciones y Estrategia Presidencial (2020) Coronavirus COVID-19 en Honduras. 1 diciembre 2020. https://covid19honduras.org

[CR5] Fromm I (2020) Agricultural Competitiveness, Sustainable Landscapes and Markets: Challenges for Honduran Agricultural Value Chains in Honduras: Economic, Political and Social Issues, Riordan, E. S. (ed), Nova Sciences Publishers, NY

[CR6] Global Hunger Index (2021) 2020 Global Hunger Index by Severity, Global Hunger Index. https://www.globalhungerindex.org/honduras.html

[CR8] Heady D, Ruel M (2020) The COVID-19 nutrition crisis: what to expect and how to protect, IFPRI issue post, published Apr. 23, 2020. https://www.ifpri.org/blog/covid-19-nutrition-crisis-what-expect-and-how-protect

[CR9] Hernandez Ore MA, Sousa LD, Lopez JH (2016). Honduras: unlocking economic potential for greater opportunities. Systematic country diagnostic.

[CR10] Instituto Nacional de Estadistica (INE) (2013) XVII Censo Nacional de Población y VI de Vivienda. http://170.238.108.227/binhnd/RpWebEngine.exe/Portal?BASE=MUNDEP10&lang=ESP

[CR11] Instituto Nacional de la Mujer (INAM) (2020) Boletín Especial COVID-19 y Género. Julio 2020. http://www.inam.gob.hn

[CR12] John Hopkins Coronavirus Resource Center (2020) Global cases by the center for systems science and engineering 2020 [dec 1 2020]. https://coronavirus.jhu.edu/map.html.

[CR13] Melvin SC, Wiggins C, Burse N, Thompson E, Monger M (2020). The role of public health in COVID-19 emergency response: efforts from a rural health perspective. Prev Chronic Dis.

[CR14] Reardon T, Bellemare M, Zilberman M (2020) How COVID-19 may disrupt food supply chains in developing countries, IFPRI Guest Post, Published Apr. 2, 2020. https://www.ifpri.org/blog/how-covid-19-may-disrupt-food-supply-chains-developing-countries

[CR15] Reiche A (2020) Mujeres en las redes agrícolas: su contribución a la agricultura sostenible (maestría). Escuela Agrícola Panamericana Zamorano, Francisco Morazán, Honduras

[CR16] ReliefWeb (2020) Small-scale farmers and families struggle with economic ruin as up to 70% of Honduras’ crops and grains are affected by storms Eta and Iota. News and Press Release, Nov. 21, 2020. https://reliefweb.int/report/honduras/small-scale-farmers-and-families-struggle-economic-ruin-70-honduras-crops-and-grains

[CR17] ReliefWeb (2021) Honduras: Tormentas Tropicales Eta e Iota - Informe de Situación No. 07, Jan. 13, 2021. https://reliefweb.int/report/honduras/honduras-tormentas-tropicales-eta-e-iota-informe-de-situaci-n-no-07-al-13-de-enero

[CR18] SEDIS (2020) Secretaria de Desarrollo e Inclusión Social. A través de Sedis más de 350 mil adultos mayores y personas con discapacidad serán beneficiados con iniciativa. Nota de Prensa. http://www.sedis.gob.hn/node/5938

[CR100] Swinnen J, McDermott J (2020) Covid-19 and global food security. Agricultural Economics Society and European Association of Agricultural Economists

[CR19] UNAH, (2020a) Boletín Oficial de la Universidad Nacional Autónoma de Honduras No. 09, Octubre 2020a. https://dircom.unah.edu.hn/dmsdocument/10473-boletin-unah-009-octubre-2020a-pdf

[CR20] UNAH (2020b) Boletín Oficial de la Universidad Nacional Autónoma de Honduras No. 10, Noviembre 2020b. https://dircom.unah.edu.hn/dmsdocument/10528-boletin-unah-010-noviembre-2020b-pdf

[CR21] UN Women (2020) The Economic Impact of COVID-19 on Women in Latin America and the Caribbean, News Article, Nov. 2, 2020. https://lac.unwomen.org/en/noticias-y-eventos/articulos/2020/11/impacto-economico-covid-19-mujeres-america-latina-y-el-caribe

[CR22] UNDP (2020) Social and economic impact of the COVID-19 and policy options in Honduras March 2020. Policy Document Series Nr. 4

[CR23] Unidad Técnica de Seguridad Alimentaria y Nutricional (UTSAN) (2019) Estudio de Indicadores de Seguridad Alimentaria y Nutricional en el Corredor Seco de Honduras. Tegucigalpa M.D.C. https://utsan.scgg.gob.hn/wp-content/uploads/2019/12/INFORME-INDICADORES-SAN-2019.pdf

[CR24] Wang CC (1999). Photovoice: a participatory action research strategy applied to women's health. J Women’s Health.

[CR101] WFP (World Food Programme) (2020) Coronavirus in honduras: ‘This pandemic is going to starve us’. Stories, published Jul. 13, 2020

[CR25] World Bank (2020) Honduras: panorama general. https://www.bancomundial.org/es/country/honduras/overview

[CR26] World Directory of Minorities and Indigenous Peoples (2020) Minorities and indigenous peoples in Honduras. https://minorityrights.org/minorities/lenca/

[CR27] WHO (2020) La OMS caracteriza covid-19 como una pandemia 2020. https://www.paho.org/hq/index.php?option=com_content&view=article&id=15756:who-characterizes-covid-19-as-a-pandemic&Itemid=1926&lang=es

